# Ectopic, intra-thyroid parathyroid adenoma better visualized by deep learning enhanced choline PET/CT

**DOI:** 10.1093/qjmed/hcaf057

**Published:** 2025-02-24

**Authors:** Kevin M Bradley, Daniel R McGowan, Lee Bartley, David Scott-Coombes, John I Rees

**Affiliations:** Wales Research and Diagnostic PET Imaging Centre, Cardiff University, Cardiff, UK; Department of Medical Physics and Clinical Engineering, Oxford University Hospitals NHS FT, Oxford, UK; Department of Oncology, University of Oxford, Oxford, UK; Department of Radiology, University Hospital of Wales, Cardiff, UK; Department of Endocrine Surgery, University Hospital of Wales, Cardiff, UK; Department of Radiology, University Hospital of Wales, Cardiff, UK

Learning points for clinicians

• Choline positron emission tomography/computed tomography (PET/CT) is a sensitive technique for localizing parathyroid adenomas, particularly ectopic adenomas.
• Ectopic parathyroid adenomas may rarely be intra-thyroid.
• PET/CT image reconstruction can now be enhanced by deep learning, an AI (artificial intelligence) technique.

Imaging for the localization of parathyroid adenomas is commonly performed to permit minimally invasive parathyroidectomy and also to detect ectopic adenomas. A plethora of modalities, techniques and radiotracers have been advocated, with over 4000 such imaging publications on PubMed. However, there will always be a problem localizing very small adenomas, particularly if ‘ectopic’. Recently, choline positron emission tomography/computed tomography (PET/CT) has emerged as a sensitive technique, with a 2023 meta-analysis of 1716 patients showing a pooled patient-based sensitivity of 93.8%.^1^ The technique appears to be of particular benefit following failed surgical exploration,^2^ regarded as the most difficult patients for which to provide a cure.

**Figure 1.**
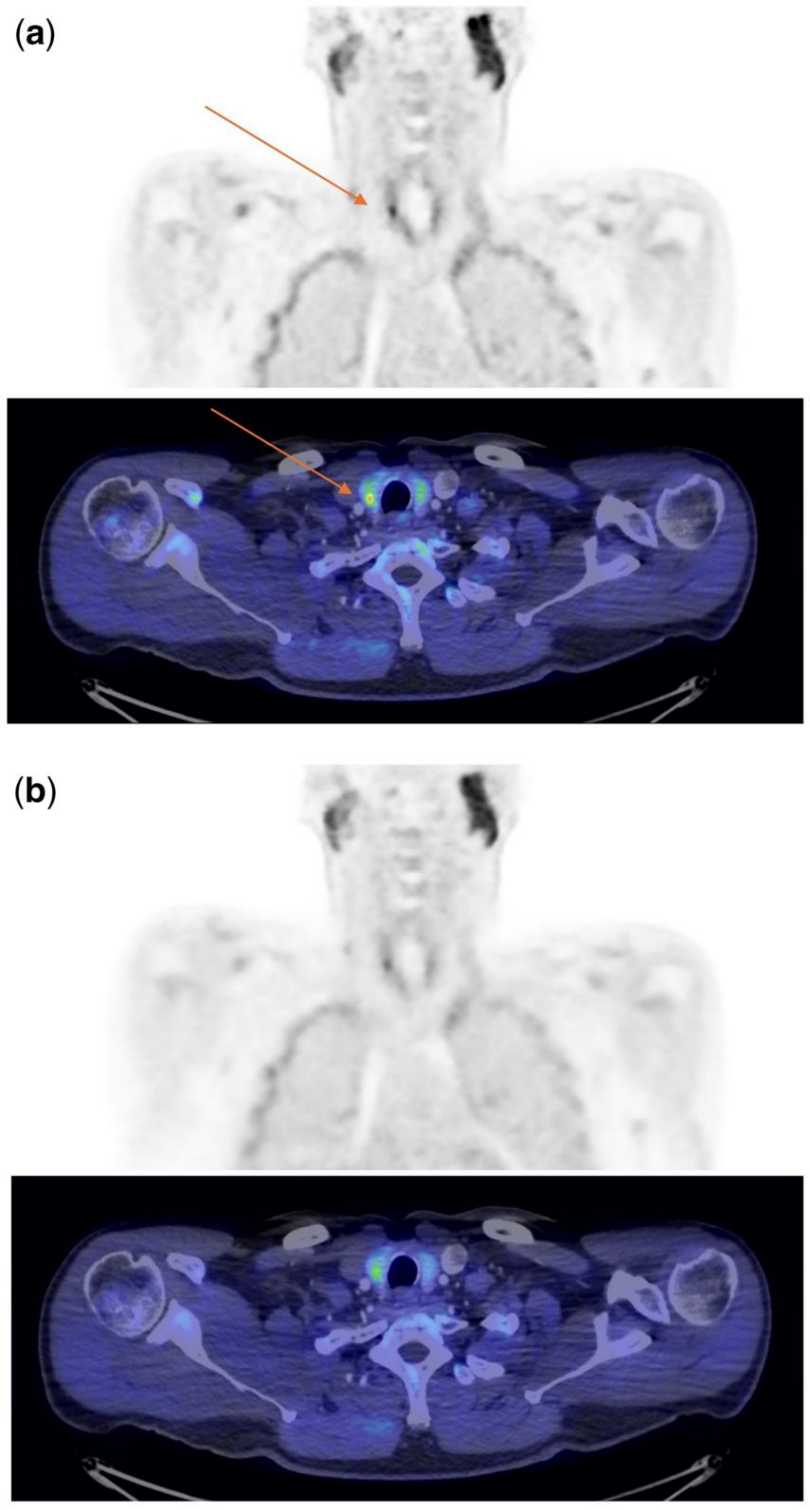
Choline coronal PET and axial PET/CT demonstrating an ectopic parathyroid adenoma within the right lobe of the thyroid, (a) better demonstrated by the deep learning enhanced PET reconstruction than (b) the standard BSREM PET reconstruction. All images on a SUV 0-6 scale.

A 60-year-old man with persistent primary hyperparathyroidism following a failed surgical neck exploration, and persistent negative ^99m^Tc-SestaMIBI and 4D-CT scans, underwent ^18^FluoroEthylCholine-PET/CT with arterial and portal phase IV contrast on a GE Omni Legend PET/CT scanner. This revealed a sub-centimetre focus of increased uptake in the right lower pole of the thyroid, more conspicuous on the deep learning enhanced PET reconstruction^3^^,^^4^ (Figure 1a, coronal PET, axial PET/CT, all images SUV 0–6) than standard BSREM (Block Sequential Regularized Expectation Maximization) PET reconstruction (Figure 1b) with associated increase in SUV_max_ (maximum standardized uptake value) from 3.1 to 4.5. Histopathology from surgical re-exploration with a right hemithyroidectomy confirmed a 6 mm parathyroid adenoma within the excised thyroid. Parathyroid hormone and calcium biochemistry were normalized at day 1, and 3 months, post-operatively.

This demonstrates the benefit of deep learning-based PET reconstruction image enhancement to reveal small foci of uptake. In this example, an elusive ectopic, intra-thyroidal parathyroid adenoma. Many such elusive adenomas are small and therefore challenging for imaging localization. Intra-thyroidal, ectopic, parathyroid adenomas are particularly difficult to locate, suggested <1% of ectopic adenomas,^5^ and if completely embedded within the thyroid, are also invisible on surgical exploration, although some may be deep within a cleft or fold from the surface of the thyroid, described as a ‘boutonnière’ adenoma. This study also supports the European Association of Nuclear Medicine guidelines^6^ which recommend that PET/CT localization should be performed on a ‘scanner with the highest system sensitivity and reconstruction protocols optimized for small lesion detection’ and provides an insight into the benefits of current developments in PET image reconstruction.
